# Awareness of health care workers with appropriate infection control practices related to multi-patient use of Close Loop Medication Administration device

**DOI:** 10.1016/j.infpip.2023.100323

**Published:** 2023-11-02

**Authors:** Kassem Abou Yassine, Aiman El-Saed, Fatmah Othman, Sarr Ramou, Bassam H. Al Alwan, Kholoud Ameer, Mustafa Hawthan, Mohammed Al Zunitan, Majid M. Alshamrani

**Affiliations:** a Infection Prevention and Control Department, King Abdulaziz Medical City, Riyadh, Saudi Arabia; b College of Public Health and Health Informatics, King Saud Bin Abdulaziz University for Health Sciences, Riyadh, Saudi Arabia; c King Abdullah International Medical Research Center, Riyadh, Saudi Arabia; d Microbiology Laboratory, King Abdullah Specialist Children Hospital, Riyadh, Saudi Arabia

**Keywords:** Infection control, Healthcare workers, Infection, Portable computerized devices, Awareness, Saudi Arabia

## Abstract

**Background:**

Portable computerized devices represent a potential source of healthcare infections. The objective was to assess the knowledge, attitudes, and practices (KAP) of healthcare workers (HCWs) toward infection control practices used with Close Loop Medication Administration (CLMA) devices. Additionally, to quantify the impact of education and training on the bacterial burden on CLMA devices.

**Methods:**

The study design consisted of two steps: a cross-sectional study was conducted among HCWs working in a tertiary care center in Riyadh, Saudi Arabia. A 32-item questionnaire was used to assess KAP information. The second step was environmental samples collected from the surfaces of CLMA devices before and after implementing a multifaceted intervention.

**Result:**

A total of 325 HCWs were included in the study. The mean age was 32.6±7.4 years. The majority were females (92%) and nurses (91.3%). The overall KAP score was 74.8%, 74.2% adequate knowledge, 79.3% positive attitude, and 71.3% appropriate practices. KAP score was better (≥ median KAP score) among HCWs working in laboratory and organ transplant units (*P*<0.001). It was also better among those with a longer duration of work experience (*P*<0.001) and those who received related training (*P*<0.001). Approximately 75% of HCWs expressed their need for more information about CLMA. Post-interventional samples had much lower bacterial burden, with the positive rate reduced from 51.4% before intervention to 16.8% after intervention (*P*<0.001).

**Conclusions:**

Awareness and behavior of HCWs about appropriate infection control practices related to portable devices is still inadequate. A multifaceted intervention including education and training significantly reduces the bioburden on portable devices.

## Introduction

Healthcare-associated infection (HAI) remains a major healthcare challenge, threatening patients' safety in healthcare settings globally [1,2]. HAI and resistant bacteria have significant impacts on healthcare resources, length of stay, and patient outcomes [1,3]. Patient care items and environmental surfaces are important potential transmission sources [4,5]. Therefore, basic practices of infection prevention and control (IPC) as well as protocols of cleaning and disinfection of the hospital environment are considered key for HAI prevention [6].

Contaminated surfaces play an important role in the transmission of HAI and antimicrobial resistance [7]. They are implicated in 20%–40% of HAIs that are transmitted by contaminated hands of HCWs [5]. Additionally, multidrug resistant bacteria (MDRO) have been retrieved from 40% to 50% of the sites examined in recently cleaned patient rooms and ICUs, respectively [8,9]. Methicillin-resistant staphylococci (MRSA) and vancomycin-resistant enterococci (VRE) were the most common pathogens cultured [8,9].

The use of portable computerized devices (such as computers on wheels, vital signs machines, portable ultrasound equipment, and other machines) are becoming very prevalent in today's healthcare services [10]. They are heavily contaminated and their use on multiple patients carries the risk of spreading HAIs [[10], [11], [12]]. A meta-analysis of 23 studies showed that more than 85% of the sampled medical equipment in healthcare settings are contaminated [13]. Nevertheless, less than 30% of HCWs were keen to regularly clean their touch screen devices [[14], [15], [16]]. Additionally, nursing cleaning of portable medical equipment may be inadequate [17,18]. Although the majority of HCWs are aware of the role of touch screen devices as a source of infection, limited cleaning knowledge and misconceptions were among reported barriers to cleaning [15,16]. The objective of the present study was to assess knowledge, attitudes, and practices (KAP) of HCWs toward IPC practices related to the use of a portable device used for drug administration called Close Loop Medication Administration (CLMA) device. This device is a new portable touch-screen device used to double-check the medications and procedures assigned to the patient. Additionally, to quantify the impact of an intervention (including education and training), on the bacterial burden on CLMA devices.

## Method

### Study design

The study design consisted of two steps: the first was a cross-sectional study conducted in April 2019 among HCWs to assess KAP toward IPC practices related to the use of CLMA devices. The second step was pre- and post-interventional assessment of a multifaceted intervention, including education and training, on bacterial contamination of these devices, using environmental samples from the surfaces of CLMA devices taken on May 2019 and again in March 2020.

### Setting

The present study has been carried out in different units/departments of King Abdulaziz Medical City, Riyadh (KAMC-R). It is an approximately 1730-bed tertiary care facility composed of two hospitals. KAMC-R provides healthcare services for almost 1.15 million eligible Saudi National Guard soldiers, employees and their families. Approximately 13% of the hospital beds in both hospitals were serving ICU patients, through 15 different ICUs. The facility is accredited by Joint Commission International (JCI). The following units/departments were included in this study: emergency department, intensive care unit, oncology/hematology, organ transplant, general pediatric wards, and laboratory.

### Sample size and sampling

Previous studies that focused on the awareness of IPC practices related to fomites including medical equipment showed variable knowledge level [14,15,19]. Therefore, we proposed an awareness level of 50% to allow for the most conservative sample size. Given the availability of 2000 HCWs in the target units/departments, it was estimated that 323 participants would be required in order to examine 50% awareness with 5% precision and 95% significance level.

### Subjects and recruitment

HCWs closely working with CLMA devices in the target units/departments including nurses and technicians were included. Physician and administrative staff were excluded. There were no exclusions based on gender or the duration of professional experience. The participants were recruited conveniently through emails, phone messages, and personal visits to the target units/departments.

### Data collection tool

An anonymous self-administered questionnaire was electronically sent to targeted HCWs. In addition to demographic and professional data, the questionnaire covered the three KAP domains of CLMA-related information. These included the pattern of use, IPC practices, and disinfection practices. They were arranged in 10 knowledge questions, 10 attitude questions, and 12 practice questions. Knowledge questions had the following responses “true”, “false”, and “don't know”. Attitude questions had the following responses; “strongly agree”, “agree”, “uncertain”, “disagree”, and “strongly disagree”. Practice questions were multiple-choice questions with variable responses. One-point was given to correct knowledge and practice questions. One-to five-points were given to increasing agreement of positive attitude. Face and content validity of the questionnaire was evaluated by infection control, infectious disease, and epidemiology professionals. The questionnaire was piloted on 15 HCWs, with very positive feedback. The questionnaire had good reliability with Cronbach's Alpha 0.76. A copy of the questionnaire is attached as supplementary material.

### Environmental sample

A pre and post-interventional study was carried out between May 2019 and March 2020. Environmental samples were collected from the surfaces of CLMA devices in May 2019 and again in March 2020. The samples were tested for bacterial contamination using standard microbiological methods. A multifaceted intervention was implemented between May 2019 and February 2020. The intervention included education and training of HCWs in target units/departments, re-defining cleaning and disinfection responsibilities of HCWs, emphasizing the auditing role of clinical resource nurse, adding a checklist and schedules for cleaning and disinfection of CLMA device, and updating of the IPC manual.

### Data analysis

Categorical variables were presented as frequencies and percentages while continuous variables were presented as means and standard deviations (SD). The overall KAP score was divided into two categories based on the median score: ≥ median and < median. Demographic and professional characteristics were compared between KAP groups. Chi-square or Fisher exact tests were used to compare categorical data while t-test was used to compare continuous data. The correlation between practices with both knowledge and attitude were done using Spearman coefficient. All *P*-values were two-tailed. A *P*-value <0.05 was considered significant. SPSS (Version 25.0. Armonk, NY: IBM Corp) was used for all statistical analyses.

## Results

### KAP scores

**Figure 1:**
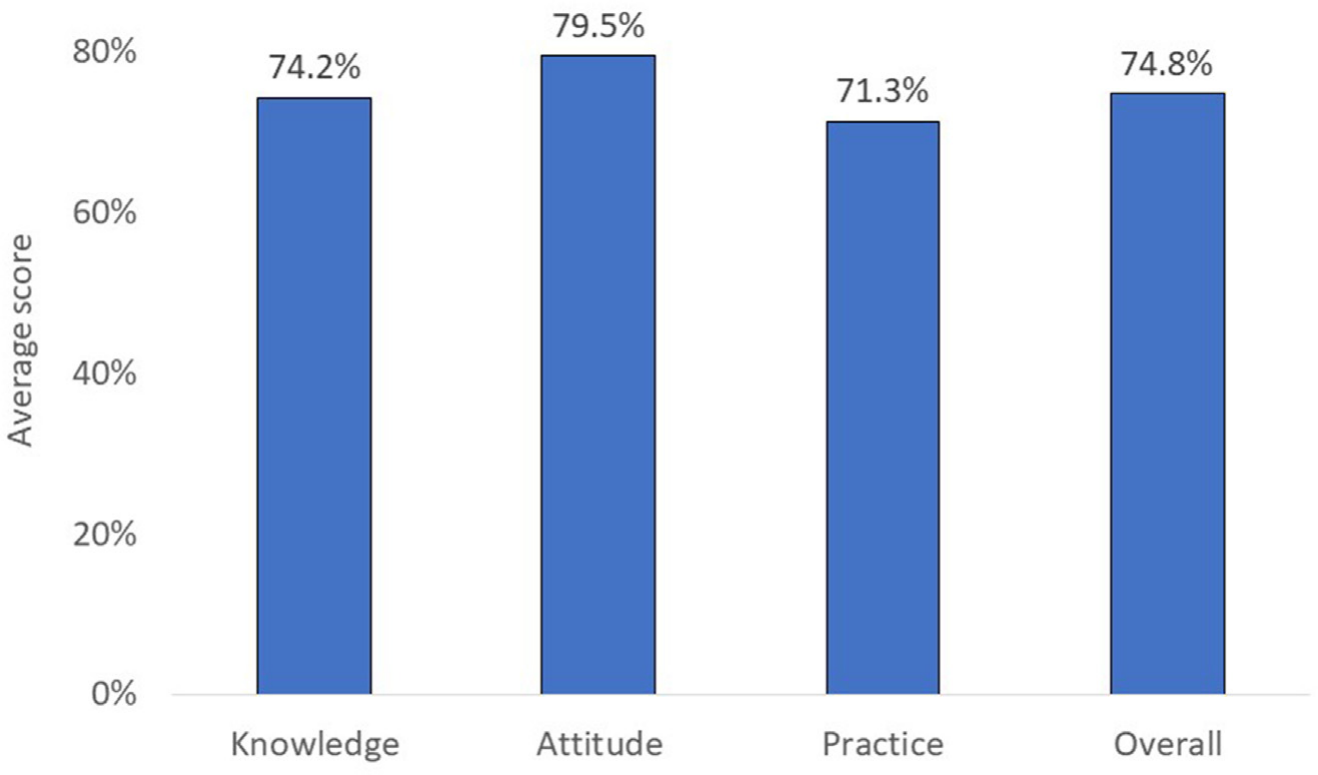
Average total and individual scores of CLMA-related knowledge, attitude, and practices before the implementation of the multifaceted intervention (CLMA, closed-loop medication administration devices).

As shown in Figure 1, the overall KAP score was 74.8%; 74.2% adequate knowledge, 79.3% positive attitude, and 71.3% appropriate practices. The details of KAP responses of the participant are shown in supplementary data (Tables 1-3).

### Demographic and professional characteristics

A total 325 HCWs were included in the study. As shown in Table I, the mean age was 32.6±7.4 years. The majority were females (92.6%), non-Saudi (90.1%), and nurses (91.3%). The average work experience was 7.9±5.2 years, with 40.1% having more than 10 years of experience, and 31% were from the intensive care unit, Table I. The most frequent work place was intensive care units (31.1%), followed by oncology/hematology wards (25.5%) and general pediatric wards (17.8%). Awareness was better (≥ median overall KAP score) in technicians compared with nurses (*P*<0.001) and in non-Saudi compared with Saudi nationals (*P*<0.001). It increased with increasing age groups (*P*<0.001), increasing duration of work experience (*P*=0.004), and higher nursing titles (*P*=0.003). Awareness was highest among those working in the laboratory (87.5%), organ transplant units (72.0%), and intensive care unit (52%) and lowest in those working in general pediatric wards (32.8%) and emergency department (41.2%).

**Table I:** Demographic characteristics of the study participants by the groups of knowledge, attitude, and practice (KAP) score before the implementation of the multifaceted intervention

	Total	KAP		*P*-value
		≥median	< Median	
Age (years)				
Mean ±SD	32.6±7.4	35.8±7.4	34.2±7.5	<0.001
<30	100 (31.8%)	32 (32.0%)	68 (68.0%)	<0.001
30-39	141 (44.9%)	78 (55.3%)	63 (44.7%)	
≥40	73 (23.2%)	47 (64.4%)	26 (35.6%)	
Gender				
Male	24 (7.4%)	14 (58.3%)	10 (41.7%)	0.405
Female	301 (92.6%)	149 (49.5%)	152 (50.5%)	
Nationality	(0.0%)	(0.0%)	(0.0%)	
Saudi	32 (9.9%)	6 (18.8%)	26 (81.3%)	<0.001
Non-Saudi	292 (90.1%)	156 (53.4%)	136 (46.6%)	
Highest educational qualification				
Diploma	94 (29.4%)	42 (44.7%)	52 (55.3%)	0.461
Bachelor	206 (64.4%)	108 (52.4%)	98 (47.6%)	
Post graduate	20 (6.3%)	10 (50.0%)	10 (50.0%)	
Work experience (years)				
Mean ±SD	7.9±5.2	10.3±6.7	9.1±6.1	<0.001
<5	73 (24.0%)	28 (38.4%)	45 (61.6%)	0.004
5-9	109 (35.9%)	48 (44.0%)	61 (56.0%)	
≥10	122 (40.1%)	74 (60.7%)	48 (39.3%)	
Professional role	(0.0%)	(0.0%)	(0.0%)	
Staff nurse 1	150 (46.4%)	63 (42.0%)	87 (58.0%)	0.003
Staff nurse 2	129 (39.9%)	66 (51.2%)	63 (48.8%)	
Senior nursing job	16 (5.0%)	10 (62.5%)	6 (37.5%)	
Technician/others	28 (8.7%)	22 (78.6%)	6 (21.4%)	
Unit				
Emergency	34 (10.5%)	14 (41.2%)	20 (58.8%)	<0.001
Intensive care unit	101 (31.1%)	53 (52.5%)	48 (47.5%)	
Oncology/hematology	83 (25.5%)	38 (45.8%)	45 (54.2%)	
Organ transplant	25 (7.7%)	18 (72.0%)	7 (28.0%)	
General pediatric	58 (17.8%)	19 (32.8%)	39 (67.2%)	
Laboratory	24 (7.4%)	21 (87.5%)	3 (12.5%)	

### CLMA-related information

As shown in Table II, the majority of HCWs acknowledged the clinical resource nurse (52.6%) and colleagues (24.6%) as the main sources of their knowledge. The majority (90.2%) received training and education about the use of CLMA devices. Approximately 54.3% of those who received CLMA-related training got cleaning and disinfection information. The majority (74.8%) expressed their need for more information about CLMA. The majority (72.4%) of HCWs who needed more information indicated that both information related to CLMA cleaning and disinfection and information related to CLMA use and operation are required. Awareness was better among HCWs who received CLMA-related training (*P*<0.001), especially training covering cleaning and disinfection (*P*=0.002) and training provided by the company (*P*=0.027) but not colleagues (0.004). Additionally, awareness was better among HCWs who did not ask for more information about CLMA (*P*=0.023).

**Table II:** CLMA-related information among the study participants by the groups of knowledge, attitude, and practice (KAP) score before the implementation of the multifaceted intervention

	Total	KAP		*P*-value
		≥median	< Median	
Source of CLMA information				
DPPs/APPs	57 (17.5%)	28 (49.1%)	29 (50.9%)	0.864
Company training	46 (14.2%)	30 (65.2%)	16 (34.8%)	0.027
Colleagues	80 (24.6%)	29 (36.3%)	51 (63.8%)	0.004
Internet	14 (4.3%)	6 (42.9%)	8 (57.1%)	0.577
Infection control manual	33 (10.2%)	17 (51.5%)	16 (48.5%)	0.869
Clinical resource nurse	171 (52.6%)	82 (48.0%)	89 (52.0%)	0.403
Infection control practitioner	24 (7.4%)	12 (50.0%)	12 (50.0%)	0.988
Others	21 (6.5%)	8 (38.1%)	13 (61.9%)	0.253
Did you receive training and education about the use of CLMA device?				
No	32 (9.8%)	5 (15.6%)	27 (84.4%)	<0.001
Yes	293 (90.2%)	158 (53.9%)	135 (46.1%)	
If yes, was cleaning and disinfection a part of the training and education?				
No	134 (45.7%)	59 (44.0%)	75 (56.0%)	0.002
Yes	159 (54.3%)	99 (62.3%)	60 (37.7%)	
Do you feel you need more information about CLMA?				
No	82 (25.2%)	50 (61.0%)	32 (39.0%)	0.023
Yes	243 (74.8%)	113 (46.5%)	130 (53.5%)	
If yes, what type of education is needed?				
Related to CLMA cleaning and disinfection	64 (26.3%)	26 (40.6%)	38 (59.4%)	0.506
Related to CLMA use and operation	3 (1.2%)	1 (33.3%)	2 (66.7%)	
Both	176 (72.4%)	86 (48.9%)	90 (51.1%)	

CLMA, closed-loop medication administration devices; APPs, administrative policies and procedures; DPPs, departmental policies and procedures.

### Correlation of practice with knowledge and attitude

As shown in supplementary data (Figure 1), there was weak positive correlation between knowledge score and practice score (r=0.141 and *P*=0.011). Additionally, there was moderate positive correlation between attitude score and practice score (r=0.275 and *P*<0.001).

### Bacterial contamination

**Figure 2:**
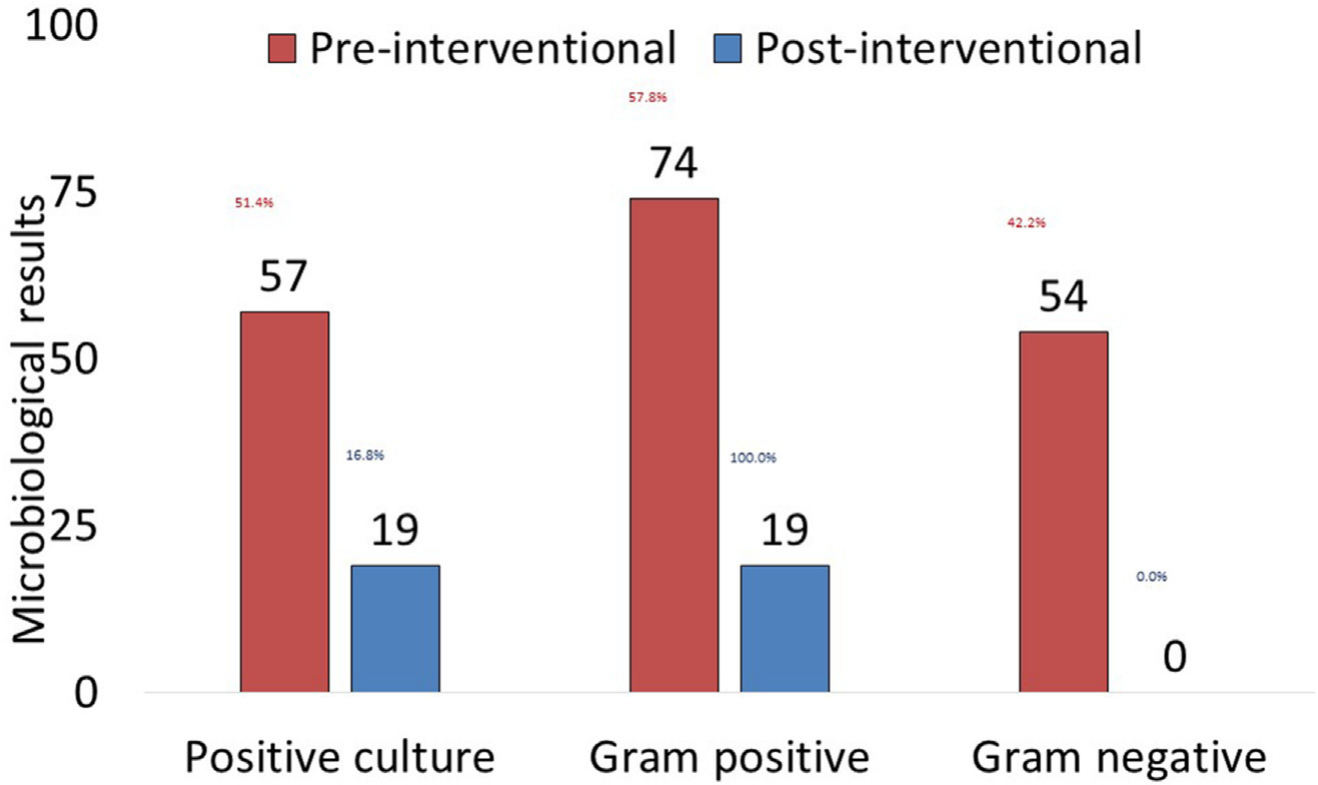
Microbiological results of samples collected from CLMA before and after the intervention.

**Figure 3:**
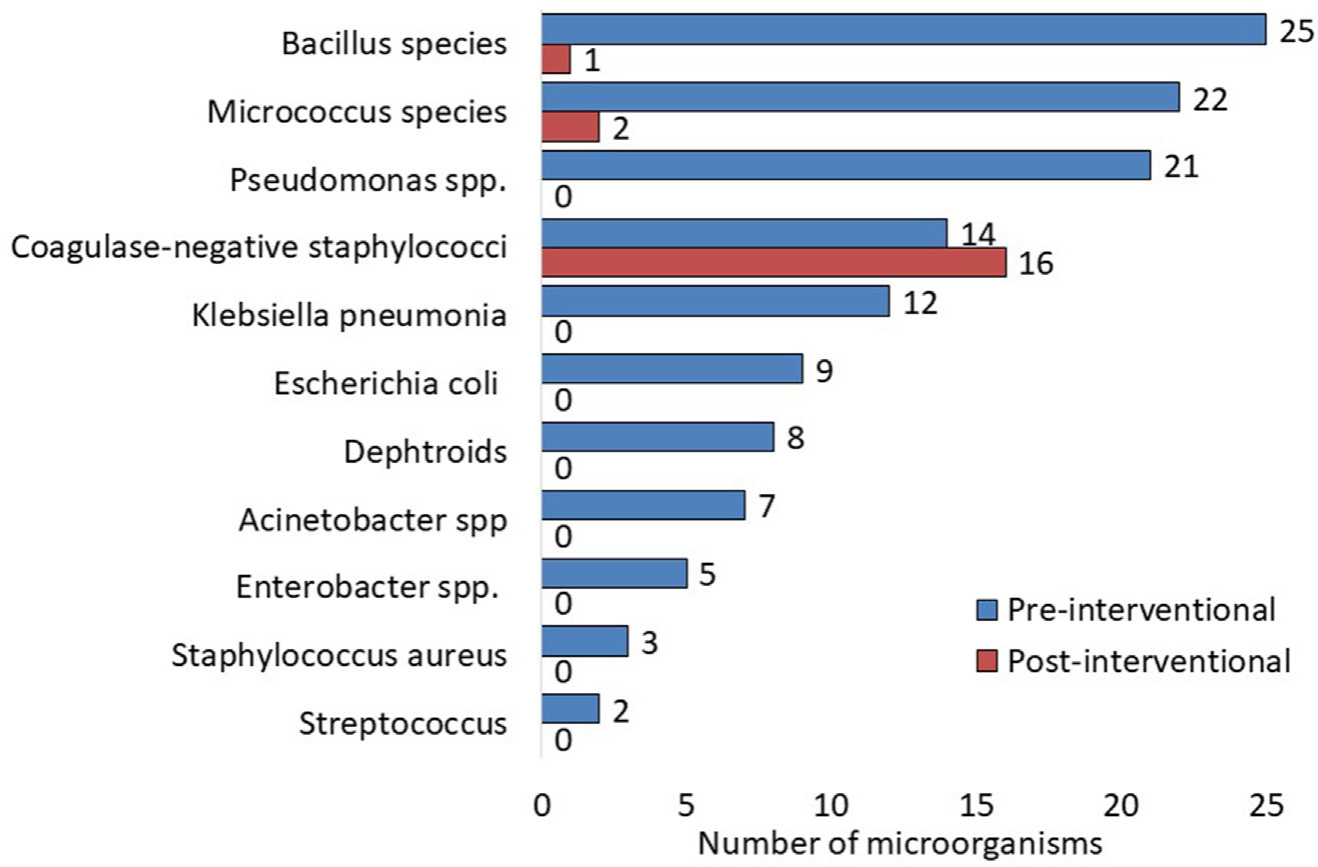
Types of bacteria detected in the samples collected from CLMA before and after the intervention.

Out of 111 CLMA devices tested in the pre-interventional assessment, 57 (51.4%) were culture positive (Figure 2). Out of 128 microorganisms grown, Bacillus species (19.5%) were the most common, followed by Micrococcus spp. (17.2%), *Pseudomonas aeruginosa* (16.4%), Coagulase-negative staphylococci (10.9%), *Klebsiella pneumoniae* (9.4%), *Escherichia coli* (7.0%), Diphtheroids (6.3%), Acinetobacter spp. (5.5%), Enterobacter spp. (3.9%), *Staphylococcus aureus* (2.3%), and Streptococcus spp. (1.6%, Figure 3). Out of 113 CLMA devices tested in the post-interventional assessment, 19 (16.8%) were culture positive with one Gram-positive microorganism (Figure 2). They included Coagulase-negative staphylococci (84.2%), Micrococcus spp. (10.5%), and Bacillus species (5.3%, Figure 3). Post-interventional samples had much lower bacterial burden, with the culture positive rate reduced from 51.4% before intervention to 16.8% after intervention (*P*<0.001), and no Gram-negative pathogens were isolated (42.2& versus 0%, *P*<0.001), Figure 2.

## Discussion

This study assessed the KAP of HCWs (mainly nursing staff) towards IPC practices related to the use of CLMA devices. The overall average KAP score was 75%, which is probably inadequate. Approximately 96% of the current participants were aware of the role CLMA devices can play in transmitting infection while 82% were reporting frequent cleaning. However, there were doubts about the reported cleaning habits. For example, 76% of HCWs were not aware of the appropriate disinfectant, 42% were unaware that the cleaning is the responsibility of all device users, and 28% were not aware that cleaning should be done before and after use of the device. Such doubts were confirmed by the relatively high (51%) pre-intervention bacterial contamination of these devices.

Comparing the current finding to existing literature is challenging due to lack of similar studies focusing on CLMA devices. Nevertheless, the current finding was consistent with previous studies that assessed awareness and behavior of HCWs, regarding cleaning and disinfection practices of hospital fomites. These studies focused on personal touch-screen devices such as mobile phones and tablets [[14], [15], [16]] and portable multiuse patient equipment such stethoscopes, blood pressure cuffs, thermometers, and glucometers [[17], [18], [19], [20]]. While almost all HCWs were aware of the importance of touch-screen devices as a source of infection, cleaning practices were poor [14,15]. For example, the prevalence of regular cleaning was 20% in a mixed group of HCWs [14], 12% in surgical teams [15], and 28% in ICU staff [16]. The reasons given for this low cleaning habits included lack of awareness of cleaning methods, lack of cleaning resources, lack of related policy, and fear of cleaning-related device damage [15,16]. On the other hand, portable multiuse patient equipment received much less perceived risk of infection and cleaning practices. For example, 45%–75% of HCWs acknowledged the role portable multiuse patient equipment play in transmitting infection [19,20] but much fewer (13%–21%) were actually cleaning them [[17], [18], [19], [20]].

As expected, awareness and appropriate practices in our study was better in more experienced HCWs. For example, higher KAP scores were associated with longer duration of work experience, older age, and higher nursing titles. Previous studies showed that more experienced healthcare workers, specially nurses, are more likely to have better knowledge of and adherence with IPC practices [21]. The longer duration of work experience is usually associated with more exposure to IPC knowledge, guidelines, audits, and training. Additionally, the awareness and practices in the present study were variable by working location, probably due to differential perceived risk [22,23]. Therefore, they were higher in high-risk locations such as laboratory and ICUs and lower in low-risk locations such as pediatric wards. Previous studies showed that higher perceived risk of infection is associated with better engagement in protective behaviors and preventative activities [22,23]. Consistently, higher attitude and knowledge scores in the present study positively correlated with appropriate cleaning practice.

As expected, receiving CLMA-related training in this study was associated with better awareness and cleaning practices. Similarly, previous studies were consistent on the positive impact of education and training on cleaning practices [17,24,25]. For example, education combined with audit and feedback were associated with increased compliance with using ultraviolet cleaning of *Clostridium difficile* rooms from 20% to 100% [24]. Consistently, a multifaceted intervention including education and training significantly reduces the bioburden on CLMA devices in the present study. Despite the 75% overall KAP score in this study, more than 70% of the HCWs expressed a wish to get more information about CMLA cleaning and disinfection. This may be the main reason behind the successful outcome of the study intervention.

The multifaceted intervention in this study was very successful in reducing the bio-burden of CLMA devices from 51% to 17%. The pre-intervention contamination is not surprising as previous studies reported bacterial growth in more than 85% of portable patient equipment, such as stethoscopes, otoscopes, and diagnostic ultrasound [13]. However, it indicates that claimed cleaning practices (as per the KAP survey) were overestimated and/or not effective. Additionally, the finding indicates an intervention that includes education, training, auditing, engagement, process adjustment, and manual update can significantly improve the CLMA-related cleaning practices. Thus, to ensure the engagement of the HCWs in the policy and to maintain the cleaning practices, the current study finding regarding the bacterial contamination pre-and post the intervention was disseminated to the HCWs and their respective departments. Thus, after completing the study, the CLMA cleaning and disinfection manual has been updated with adjustments in recommendations. Those adjustments include a detailed process to clean and disinfect CLAM devices with approved disinfection products.

Bacterial examination before and after cleaning has been used by multiple studies in assessing the efficacy of cleaning methods [[26], [27], [28]]. For example, cleaning of personal digital assistants of HCWs using different disinfectant swabs significantly reduces bacterial contamination from 96% to 8%–44% [27,28]. It should be noticed that cleaning of CLMA in the present study eliminated all pathogenic bacteria but was not efficient for some skin commensals such as Coagulase-negative staphylococci [26,29]. This may indicate that the benefit of cleaning can be maximized by following other IPC practices, especially hand hygiene [30].

Our study has some limitations. As the study used a cross-sectional design to assess CLMA-related awareness and practices, bias cannot be excluded, and causality cannot be proved. Additionally, the studied HCWs were recruited from a single institution which may limit the generalizability of the findings. Despite those limitations, this study investigated the reported cleaning practices using environmental samples. The combined study design allowed assessment of the impact of a multifaceted intervention. Finally, the intervention did not focus only on education and training but also monitoring, auditing, engagement, process adjustment, and manual update.

In conclusion, awareness and behavior of IPC practices related to the use of CLMA devices were assessed in a group of HCWs. The overall KAP score was 75%, including 74% adequate knowledge, 79% positive attitude, and 71% appropriate practices. Better awareness and practices were associated with older age, longer duration of work experience, higher nursing titles, and receiving related training. A multifaceted intervention including education, training, auditing, engagement, process adjustment, and manual update significantly improved the CLMA-related cleaning practices. This was confirmed by reduction of positive bacteria cultures from 51% in pre-intervention samples to 17% post-intervention samples, with removal of pathogenic bacteria.

## Credit author statement

Kassem Abou Yassine, Aiman El-Saed, Majid M. Alshamrani conceived the study idea. Kassem Abou Yassine; Aiman El-Saed, Majid M. Alshamrani, Sarr Ramou, Bassam H. Al Alwan, Kholoud Ameer, Mustafa Hawthan,and Mohammed Al Zunitan were involved in the methodology, data collection, Data curation, and analysis plan. Aiman El-Saed and Fatmah Othman analyzed the data. Majid M. Alshamrani,Kassem Abou Yassine; Aiman El-Saed, Sarr Ramou, Bassam H. Al Alwan, Kholoud Ameer, Mustafa Hawthan, Fatmah Othman and Mohammed Al Zunitan were involved in visualization, investigation, and validation of the study. All authors contributed to the first draft of the manuscript and critically revised the manuscript. All authors contributed to the drafting and editing of the final manuscript and have provided their permission to publish the manuscript. All authors agree to take responsibility for the work.

## Ethics approval

The study obtained all required approvals from the IRB committee of King Abdullah International Medical Research Center (KAIMRC), Riyadh, Saudi Arabia. protocol number NRC23R/248/03.

## Funding source

The authors received no financial support related to this research.

## Conflict of interest

All authors have no known competing financial interests or personal relationships that could have appeared to influence the work reported in this paper.
