# Supplementary material for: Awareness of health care workers with appropriate infection control practices related to multi-patient use of Close Loop Medication Administration device
Source: Infect Prev Pract. 2023 Nov 2;5(4):100323. doi: 10.1016/j.infpip.2023.100323 (PMC10665828; doi:10.1016/j.infpip.2023.100323)
Supplement: Multimedia component 1 [file mmc1.docx]

**Supplement Table 1: Responses of the study participants to CLMA-related knowledge questions**

|  | **True** | **False** | **Don’t know** | **Correct answer** |
| --- | --- | --- | --- | --- |
| CLMA device can be a tool for germ transmission | 310 (96.0%) | 9 (2.8%) | 4 (1.2%) | 310 (96.0%) |
| Frequent cleaning and disinfection of the CLMA device can reduce the risk of germ transmission | 306 (94.4%) | 6 (1.9%) | 12 (3.7%) | 306 (94.4%) |
| There is no relation between the transmission of infection and the CLMA device because it is not touching the patient directly | 30 (9.3%) | 263 (81.2%) | 31 (9.6%) | 263 (81.2%) |
| There is a specific cleaning and disinfection manual for the CLMA device | 126 (39.3%) | 76 (23.7%) | 119 (37.1%) | 126 (39.3%) |
| The CLMA device is compatible to all the cleaning and disinfectant solutions | 77 (23.9%) | 103 (32.0%) | 142 (44.1%) | 103 (32.0%) |
| Some health care associated infections are preventable by following the infection control measures | 299 (92.0%) | 17 (5.2%) | 9 (2.8%) | 299 (92.0%) |
| Spore forming germs can be easily killed by using any cleaning solution | 72 (22.2%) | 229 (70.7%) | 23 (7.1%) | 229 (70.7%) |
| There is no policy for cleaning and disinfection of equipment in your hospital | 34 (10.6%) | 251 (78.2%) | 36 (11.2%) | 251 (78.2%) |
| The CLMA device need to be cleaned before and after patient care | 309 (96.6%) | 6 (1.9%) | 5 (1.6%) | 309 (96.6%) |
| The contact time of all disinfectant used is the same | 92 (28.6%) | 199 (61.8%) | 31 (9.6%) | 199 (61.8%) |

CLMA, closed-loop medication administration devices

**Supplement Table 2: Responses of the study participants to CLMA-related attitude questions**

| Do you think/believe… | **Strongly agree** | **Agree** | **Uncertain** | **Disagree** | **Strongly disagree** |
| --- | --- | --- | --- | --- | --- |
| Hand hygiene is needed between scanning patient's barcode | 149 (45.8%) | 128 (39.4%) | 17 (5.2%) | 17 (5.2%) | 14 (4.3%) |
| Performing hand hygiene after the patient's barcode scanning reduces germ transmission risk | 129 (39.8%) | 162 (50.0%) | 18 (5.6%) | 12 (3.7%) | 3 (0.9%) |
| If you are wearing gloves, hand hygiene is still needed after the barcode scanning | 130 (40.0%) | 148 (45.5%) | 19 (5.8%) | 19 (5.8%) | 9 (2.8%) |
| Frequent disinfection of the CLMA is necessary because it is touching the patient directly | 85 (26.2%) | 176 (54.3%) | 37 (11.4%) | 25 (7.7%) | 1 (0.3%) |
| There is enough time to clean and disinfect the CLMA device frequently, even with other patient care responsibilities | 54 (16.6%) | 152 (46.8%) | 42 (12.9%) | 58 (17.8%) | 19 (5.8%) |
| If you are wearing gloves, you still need to clean or disinfect the CLMA device because it could be contaminated | 111 (34.2%) | 185 (56.9%) | 12 (3.7%) | 15 (4.6%) | 2 (0.6%) |
| Allowing the disinfectant to dry can help in preventing infection transmission | 80 (24.8%) | 168 (52.0%) | 38 (11.8%) | 28 (8.7%) | 9 (2.8%) |
| Cleaning of the CLMA device should not be assigned to the patient care technicians or housekeeping staff only | 85 (26.2%) | 170 (52.3%) | 42 (12.9%) | 19 (5.8%) | 9 (2.8%) |
| Cleaning and disinfection of CLMA is the same whether in isolation or non-isolation rooms | 74 (22.9%) | 137 (42.4%) | 52 (16.1%) | 46 (14.2%) | 14 (4.3%) |
| Inappropriate cleaning and disinfection of the CLMA can lead to a cluster or outbreak of infectious disease | 101 (31.2%) | 169 (52.2%) | 30 (9.3%) | 17 (5.2%) | 7 (2.2%) |

CLMA, closed-loop medication administration devices

**Supplement Table 3: Responses of the study participants to CLMA-related practice questions**

| **Practice question** | **Value** | **Practice question** | **Value** |
| --- | --- | --- | --- |
| **How often do you perform hand hygiene after scanning the patient's barcode?** |  | **How often do you wear gloves before using the CLMA device** |  |
| Never | 2 (0.6%) | Never | 8 (2.5%) |
| Sometimes | 40 (12.3%) | Rarely | 36 (11.1%) |
| Often | 34 (10.5%) | Often | 112 (34.7%) |
| Always* | 249 (76.6%) | Always* | 167 (51.7%) |
| **Who is responsible to clean the CLMA device in you unit?** |  | **When do you perform cleaning and disinfection of the CLMA** |  |
| Patient care technician | 11 (3.4%) | Before patient care | 8 (2.5%) |
| Housekeeper | 1 (0.3%) | After patient care | 39 (12.0%) |
| Phlebotomist | 4 (1.2%) | Before and after patient care* | 234 (72.0%) |
| Nurses | 114 (35.1%) | Once per shift | 40 (12.3%) |
| Any user* | 187 (57.6%) | At night only | 4 (1.2%) |
| Don't know | 10 (3.1%) | **Who is using the CLMA?** |  |
| **Is the CLMA device used for** |  | Patient care technician | 4 (1.2%) |
| Single patient | 81 (25.1%) | Housekeeper* | 1 (0.3%) |
| Multiple patients* | 242 (74.9%) | Phlebotomist* | 45 (13.7%) |
| **Do you bring the CLMA inside the isolation room?** |  | Nurses | 282 (86.7%) |
| No | 21 (6.5%) | Doctors | 5 (1.5%) |
| Yes* | 302 (93.5%) | All of the above | 13 (4.0%) |
| **What do you usually use for hand hygiene?** |  | **How often do you disinfect the CLMA device before patient care** |  |
| Alcohol-based hand rub only | 54 (16.6%) | Never | 8 (2.5%) |
| Soap and water only | 4 (1.2%) | Rarely | 49 (15.1%) |
| Both* | 266 (81.8%) | Often | 95 (29.3%) |
| Other | 1 (0.3%) | Always* | 172 (53.1%) |
| **Do you allow the disinfectant to dry before you can use the CLMA device?** |  | **What do you use to clean and disinfect the CLMA device** |  |
| No | 27 (8.3%) | Chlorine bleach 5% wipes* | 2 (0.6%) |
| Yes* | 297 (91.7%) | Alcohol 70% swabs | 56 (17.3%) |
| **For what type of procedure do you use the CLMA?** |  | 3M Quat disinfectant cleaner concentrate (5H) | 26 (8.0%) |
| Medication and infusion administration | 5 (1.5%) | Medipal Alcohol 70% disinfectant wipes | 93 (28.7%) |
| Total parenteral nutrition | 0 (0.0%) | Medipal 20% Chlorhexidine in 70% alcohol wipes* | 77 (23.8%) |
| Blood & blood product transfusion | 0 (0.0%) | Others | 70 (21.6%) |
| Laboratory sampling | 23 (7.1%) |  |  |
| All of the above* | 297 (91.4%) |  |  |

* Correct answer; CLMA, closed-loop medication administration devices

| 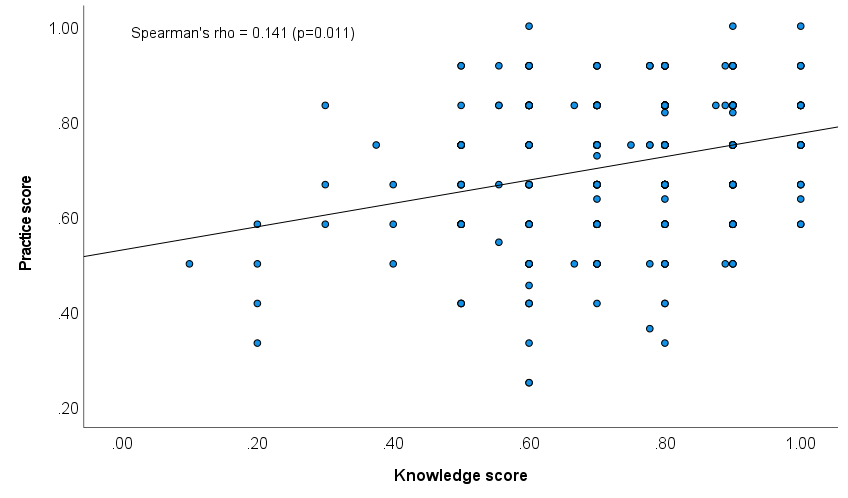 |
| --- |
|  |
| 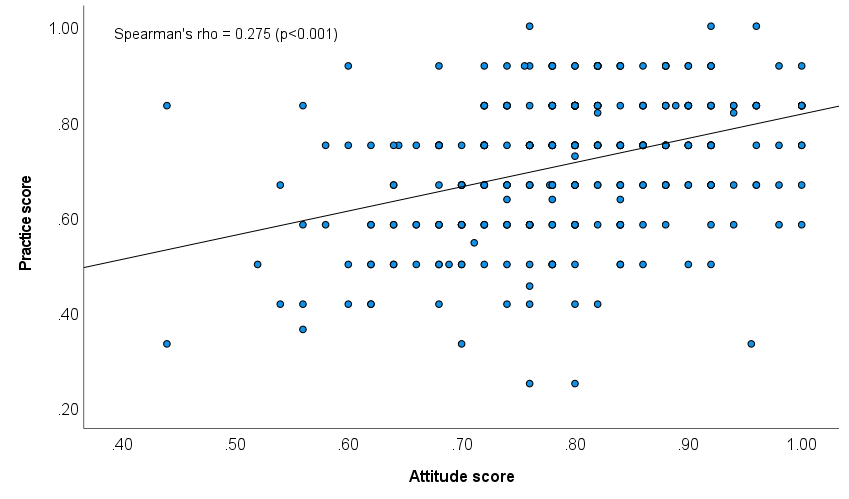 |

**Supplement Figure 1: Correlation of practice score with knowledge score (above) and attitude score (below) among the study participants**
